# Evidence for genetic regulation of mRNA expression of the dosage-sensitive gene *retinoic acid induced-1* (*RAI1*) in human brain

**DOI:** 10.1038/srep19010

**Published:** 2016-01-08

**Authors:** Li Chen, Yu Tao, Fan Song, Xi Yuan, Jian Wang, David Saffen

**Affiliations:** 1 Department of Cellular and Genetic Medicine, School of Basic Medical Sciences, Fudan University, Shanghai, China; 2 Institutes of Brain Science, Fudan University, Shanghai, China; 3 State Key Laboratory for Medical Neurobiology, Fudan University, Shanghai, China; 4 Key Laboratory of Exploration and Utilization of Aquatic Genetic Resources, Shanghai Ocean University, Shanghai, Ministry of Education, China

## Abstract

*RAI1* (*retinoic acid induced-1*) is a dosage-sensitive gene that causes Smith-Magenis syndrome (SMS) when mutated or deleted and Potocki-Lupski Syndrome (PTLS) when duplicated, with psychiatric features commonly observed in both syndromes. How common genetic variants regulate this gene, however, is unknown. In this study, we found that *RAI1* mRNA expression in Chinese prefrontal and temporal cortex correlate with genotypes of common single nucleotide polymorphisms (SNPs) located in the *RAI1* 5′-upstream region. Using genotype imputation, “R^2^-Δ^2^” analysis, and data from the RegulomeDB database, we identified SNPs rs4925102 and rs9907986 as possible regulatory variants, accounting for approximately 30–40% of the variance in *RAI1* mRNA expression in both brain regions. Specifically, rs4925102 and rs9907986 are predicted to disrupt the binding of retinoic acid RXR-RAR receptors and the transcription factor DEAF1 (Deformed epidermal autoregulatory factor-1), respectively. Consistent with these predictions, we observed binding of RXRα and RARα to the predicted *RAI1* target in chromatin immunoprecipitation assays. Retinoic acid is crucial for early development of the central neural system, and *DEAF1* is associated with intellectual disability. The observation that a significant portion of *RAI1* mRNA expression is genetically controlled raises the possibility that common *RAI1* 5′-region regulatory variants contribute more generally to psychiatric disorders.

*Retinoic acid induced 1 (RAI1)* is a dose-sensitive gene located at Chr17p11.2, an unstable region subject to recurrent microdeletions, deletions, and duplications resulting from non-allelic homologous recombination (NAHR) between low-copy repeats (LCRs)^1^. Deletions of the chromosomal segment that includes *RAI1* cause Smith-Magenis syndrome (SMS)^2^, while duplications of the same chromosomal segment cause Potocki-Lupski Syndrome (PTLS)^3^. SMS, which has an estimated prevalence of one in 15,000 to 25,000 live births, is characterized by craniofacial and cardiovascular anomalies, developmental delay, sleep disturbances, obesity, intellectual impairment, and self-injurious, aggressive and autistic-like behaviors^2^. PTLS, which is a less severe syndrome and therefore possibly less frequently diagnosed, is characterized by failure-to-thrive, developmental delay, mild-to-severe intellectual impairment, hyperactivity and autistic behaviors^3^.

The identification of rare nonsense and frameshift mutations in *RAI1* that reproduce many of the features of SMS in the absence of Chr17p11.2 deletions, strongly implies that *RAI1* haploinsufficiency is the primary cause of the syndrome^4^,^5^. This hypothesis is supported by murine models of SMS, where deletion of a 2 Mb region containing the murine *Rai1* gene^6^,^7^ or targeted inactivation of the *Rai1* gene^8^,^9^ reproduce many of the clinical phenotypes observed in SMS patients, including craniofacial abnormalities, seizures, abnormal circadian rhythm, and obesity.

Evidence that *RAI1* over-expression is the primary cause of PTLS is supported by the observations that: i) heterogeneous duplications that give rise to this disorder all include *RAI1* and ii) a 125 kb “smallest region of overlap” for these deletions contains only *RAI1*^10^. Studies using murine models of PTLS are also consistent with this conclusion. Mice harboring a 2 Mb duplication including *Rai1* exhibit low body weight, hyperactivity, learn and memory deficits and abnormal maternal behavior^6^,^7^ and transgenic mice containing two extra copies of *Rai1* show abnormal early post-natal development, hyperactivity, anxiety-related behaviors, and abnormal maternal behavior^11^. Taken together, the symptoms of SMS and PTLS patients and the phenotypes of mice harboring *Rai1* deletions or duplications imply that *RAI1* functions in multiple pathways related to development and neurological function in a dosage-dependent manner.

*Rai1* (originally named *Gt1*) was first identified as a gene induced in the mouse embryonal carcinoma cell line P19 by retinoic acid, a treatment that causes the cells to differentiate into neurons and glia^12^. The human *RAI1* gene^13^,^14^, which is highly homologous to mouse *Rai1*, spans 130 kb and contains 6 exons. The encoded 1906 amino acid-containing protein localizes to the cellular nucleus^15^ and is widely expressed in tissues throughout the body, with particularly high levels in brain and heart^14^. In the mouse brain, RAI1 protein is expressed primarily in neurons, including pyramidal cells of the hippocampus, granule cells of the dentate gyrus, neurons in the neocortex, and Purkinje cells of the cerebellum^8^. RAI1 protein was recently shown to be expressed at high levels in neurons, but not glia, of the dentate gyrus and CA layer of the hippocampus of human brain^16^. *RAI1* mRNA has been detected in human frontal and temporal lobes by northern blot analysis^13^.

Bioinformatics analysis^17^ and gene expression assays^9^,^15^ suggest that RAI1 directly or indirectly influences transcription. RAI1 contains a PHD (plant homeodomain) motif in the carboxyl terminal region that is homologous to a PHD domain in the transcriptional co-activator TCF20 (AR1/SPBP)^18^. A comparative study of SPBP and RAI1 suggests that both proteins may function as “histone readers,” proteins that recognize specific post-translational modifications of histones and simultaneously serve as platforms for the recruitment of proteins that regulate DNA metabolic activities, including RNA transcription^19^. The presence of a bipartite nuclear localization signal^1^ in RAI1 is consistent with a role in the regulation of gene expression. A polyglutamine domain in the N-terminal domain resembles those found in many transcription factors^20^.

Because *RAI1* is a dosage-sensitive gene, we wondered whether there are common regulatory variants that could potentially function as genetic modifying factors for SMS or PTLS or contribute more generally to neuropsychiatric disorders. In this study, we identified SNPs rs9907986 and rs4925102, which are located in the *RAI1* 5′-region within predicted binding sites for the transcription factors DEAF1 and RXRα-RARα, respectively, as possible regulatory variants for *RAI1* mRNA expression in prefrontal cortex and temporal cortex. Because retinoic acid is crucial for early development of the central nervous system, and mutations in *DEAF1* are associated with intellectual disability, genetic variants in *RAI1* that influence the function of these upstream factors may contribute to phenotypic differences in SMS and PTLS, and, possibly, other neuropsychiatric disorders.

## Results

### Identification of quantitative trait loci within the *RAI1* 5′-region

**Figure 1:**
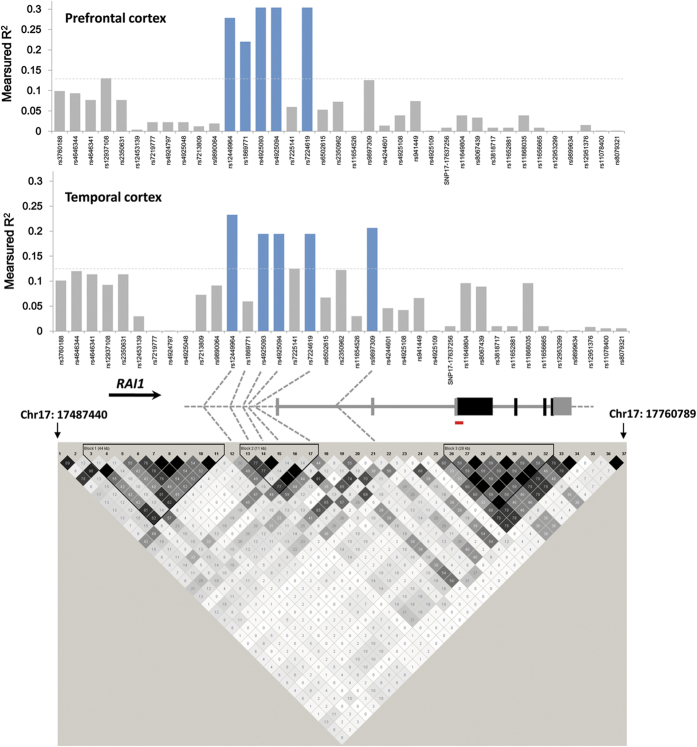
Identification of genotyped SNPs within the *RAI1* locus that correlate with mRNA expression in prefrontal cortex and temporal cortex. (*top*) Plots of coefficients of determination (R^2^) from linear regression analysis of *RAI1* mRNA expression in prefrontal cortex (upper panel) and temporal cortex (lower panel) vs. genotypes for 37 SNPs located within the neighborhood (~273 kb) of the *RAI1* gene. SNPs exceeding the threshold for nominal statistical significance (i.e., P < 0.05, uncorrected for multiple testing) are indicated by blue bars. (P = 0.05 thresholds denoted by horizontal dotted lines.) (*middle*) Model of the *RAI1* gene showing its intron/exon structure, direction of transcription and locations of key SNPs. The red bar indicates the location of the PCR product amplified from the cDNA used to quantify *RAI1* mRNA expression. (*bottom*) Plot of pairwise Δ^2^ ( = r^2^) LD constants based on genotype data for Han Chinese individuals in our collection generated using Haploview^56^. Dark gray boxes: SNP pairs with 0.9 < Δ^2^ < 1; various shades of gray squares (from light to dark): 0.02 < Δ^2^ < 0.9; white squares: 0 < Δ^2^ < 0.02. Numbers at each end of the triangle indicate the locations of the chromosome segment analyzed (based on GRCh37hg 19 coordinates).

To identify common regulatory genetic variants within the *RAI1* locus, we scanned 37 genotyped SNPs within a ~273 kb chromosomal segment containing the *RAI1* gene for correlations with *RAI1* mRNA levels in samples of Han Chinese prefrontal cortex and temporal cortex. This analysis revealed a set of common SNPs located within the 5′-upstream region of the gene that showed nominally significant correlations between genotype and *RAI1* mRNA expression in both brain regions (Fig. 1). To identify “low-” and “high-expression” alleles, we plotted normalized *RAI1* mRNA expression (in ΔΔC_t_ units) in prefrontal and temporal cortex as a function of genotype for each SNP (Supplementary File, Fig. S1). Assignments of “low-” and “high-expression” alleles for each SNP and linear regression P-values for *RAI1* mRNA expression stratified by SNPs genotype are listed in Table S1.

It should be noted that SNPs for which we have genotype information represent approximately 14% of the known SNPs in this region with allele frequencies >1% (listed in dbSNP: http://www.ncbi.nlm.nih.gov/projects/SNP/). To identify potential functional SNPs within the *RAI1* region, we imputed genotypes for common SNPs using genotype data from the 1000 Genomes Project as a reference. Again, nominally significant eQTL peaks identified using the combined genotyped and imputed data were all located within the 5′-upstream region of the *RAI1* gene (Fig. S2).

### Identification of index SNPs in the eQTL of *RAI1* 5′-region

**Figure 2:**
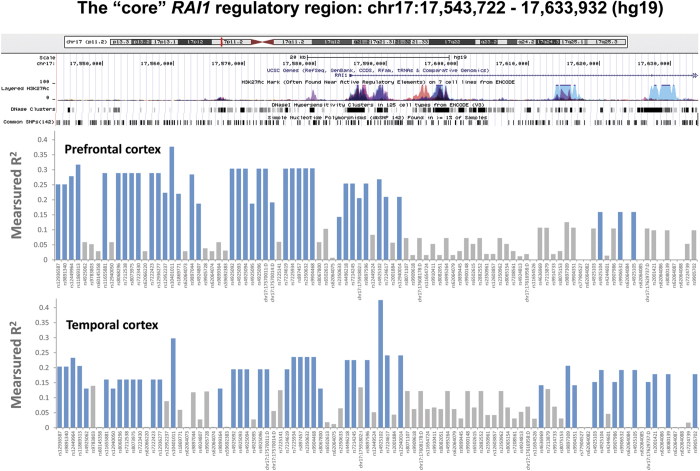
The *RAI1* “core” regulatory region. (*top*) Screen shot from the UCSC genome browser (http://genome.ucsc.edu) for the indicated region of chromosome 17, showing the locations of the 5′-end of the *RAI1* gene, histone III lysine 27 acetylation (H3K27Ac) and DNase sensitive clusters. (*bottom*) Plots of linear regression R^2^ values for *RAI1* SNPs with measured or imputed genotypes (total: 96 SNPs within 90.2 kb). SNPs with nominally significant correlations between genotype and *RAI1* mRNA expression in prefrontal cortex or temporal cortex are indicated by blue bars.

To obtain insights concerning the number of regulatory variants within the ~90 kb “core” *RAI1* 5′-region that significantly correlate with *RAI1* expression in both brain regions (Fig. 2), we constructed “R^2^-Δ^2^ plots^21^,” which group SNPs into families based on association (measured asΔ^2^) with “index” SNPs that make the largest contributions to the variance of measured coefficients of determination (R^2^) for SNPs within this region. Here we use Δ^2^ to denote the standard “r^2^” linkage disequilibrium (LD) constant, to avoid confusion with the linear regression coefficient of determination (R^2^). As described in Methods, this analysis began with the examination of all possible sets of 2 or 3 SNPs among the set of 96 genotyped or imputed SNPs (with minor allele frequencies > 0.01; missing genotype rate < 0.05; and Hardy–Weinberg equilibrium (HWE) P-values > 0.001 and pruned to eliminate SNPs with identical genotypes) to identify a specific combination of SNPs that best account for the observed set of R^2^-values associated with individual SNPs in the “core” region. The results of this analysis for 3-SNP combinations for the prefrontal cortex and temporal cortex are shown in Fig. S3. SNPs contained within the highest scoring 3-SNP combination (i.e., the combination of SNPs yielding the highest adjusted-R^2^ value in multivariable regression analysis of *RAI1* mRNA expression vs. SNP genotypes) were selected as “index” SNPs for further study.

**Figure 3:**
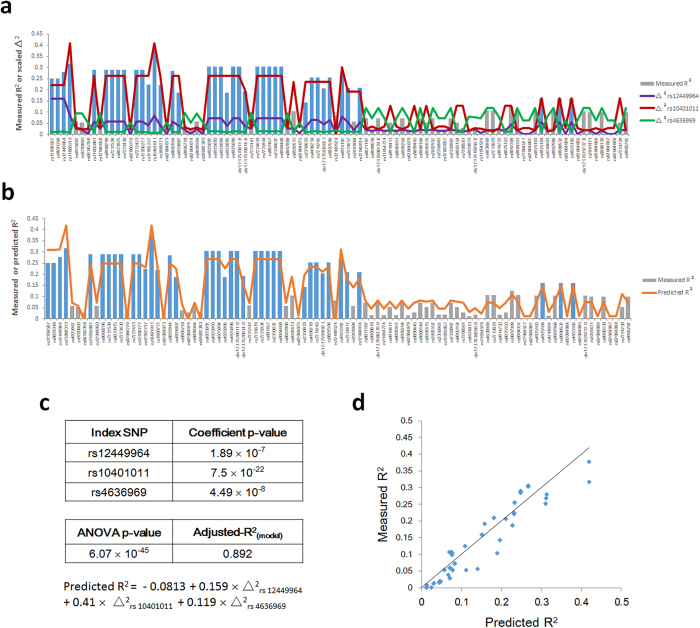
Visualization of SNP families that contribute to *RAI1* mRNA expression in prefrontal cortex. (**a**) R^2^-Δ^2^ plot showing the contributions of three SNP families associated with the “index” SNPs rs12449964, rs10401011 and rs4636969, respectively, to measured R^2^ values for 96 genotyped or imputed SNPs within the *RAI1* core regulatory region. (**b**) R^2^-R^2^ plot showing measured vs. predicted R^2^ values for the same set of SNPs. (**c**) Regression equation derived from multivariable linear regression analysis of measured R^2^ values *vs.* Δ^2^_ij_ values associated with the index SNPs rs12449964, rs10401011 and rs4636969. The variance inflation factors (VIFs) for the Δ^2^_ij_ independent variables were all < 5.0; range: 1.8–3.6 (see Methods for details). P-values for regression coefficients and the ANOVA-based P-value and adjusted R^2^_model_ for the overall fit of the model are listed in the panel. (**d**) Plot of measured *vs.* predicted R^2^-values for the model based on the index SNPs rs12449964, rs10401011 and rs4636969.

**Figure 4:**
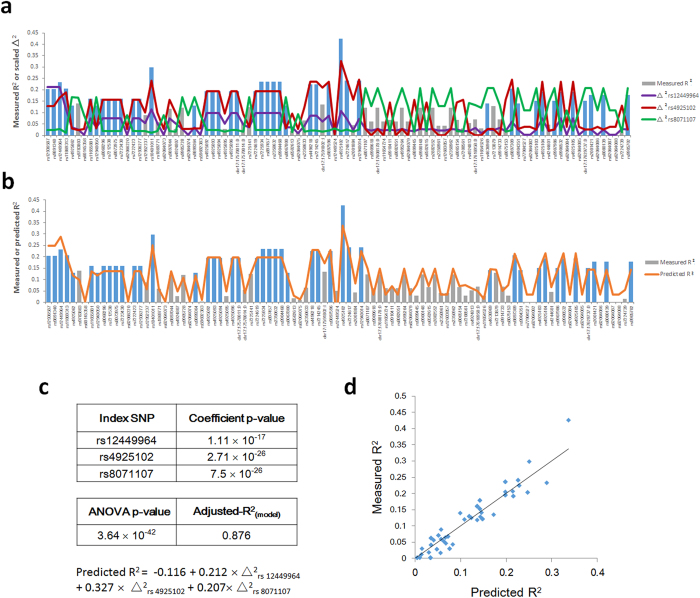
Visualization of SNP families that contribute to *RAI1* mRNA expression in temporal cortex. (**a**) R^2^-Δ^2^ plot, (**b**) R^2^-R^2^ plot (**c**) Regression equation and associated P-values and R^2^_model_, (**d**) Plot showing linear correlation between measured and predicted R^2^ values as described in the legend to Fig. 3, but based on the “index” SNPs rs12449964, rs4925102 and rs8071107. As above, the VIF associated with each Δ^2^_ij_ was < 5.0.

As shown in Fig. 3A, grouping *RAI1* SNPs according to association with the “index” SNPs rs12449964, rs10401011 and rs4636969 (identified as described above) accounts for most of the measured R^2^ values associated with SNPs in the *RAI1* 5′-region for mRNA expression in prefrontal cortex. Figure 3B shows a plot of the regression line (i.e., predicted R^2^ values) obtained from this analysis superimposed on the measured R^2^ values associated with *RAI1* 5′-region SNPs. By inspection, the regression line can be seen to account for most of the measured R^2^ values. Figure 3C shows the statistical analysis of this linear regression, which yielded highly significant P-values for the regression coefficients and overall fit (ANOVA) and an adusted-R^2^_(model)_ of 0.892. Figure 3D shows a plot of measured R^2^-values *vs.* the predicted R^2^-values calculated using the regression equation. As shown in Fig. 4, a similar analysis of *RAI1* mRNA in temporal cortex identified SNPs rs12449964, rs4925102 and rs8071107 as the index SNPs, with highly significant P-values and an adjusted-R^2^_(model)_ = 0.876.

### Identification of putative functional SNPs from the two index SNPs families in *RAI1* 5′-region

To determine whether *RAI1* mRNA expression is regulated by the same functional SNPs in the two brain regions, we investigated SNPs that are in LD (Δ^2^ > 0.5) with each of the three index SNPs in the prefrontal cortex or temporal cortex samples. This analysis showed that the index SNPs rs4636969 and rs8071107 are in complete LD and SNP rs10401011 and rs4925102 are associated with a Δ^2^ LD constant of 0.735 (Table 1).

To identify possible functional SNPs, we used RegulomeDB (http://regulomedb.org/) to screen lists of SNPs from the 1000 Genome Project data base that are in LD with our index SNPs, selecting SNPs with RegulomeDB scores 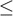
2 for further investigation. A RegulomeDB score of 1 signifies that the SNP: i) has been identified as an eQTL and there is also experimental evidence for: ii) the binding of a transcription factor (TF) and iii) a matched TF binding motif, and/or iv) a matched DNase footprint, and/or v) co-localization with a DNase-sensitive site. A RegulomeDB score of 2 signifies that eQTL evidence is lacking, but there is evidence for: i) the binding of a TF and one or more of properties iii-v listed above.

**Figure 5:**
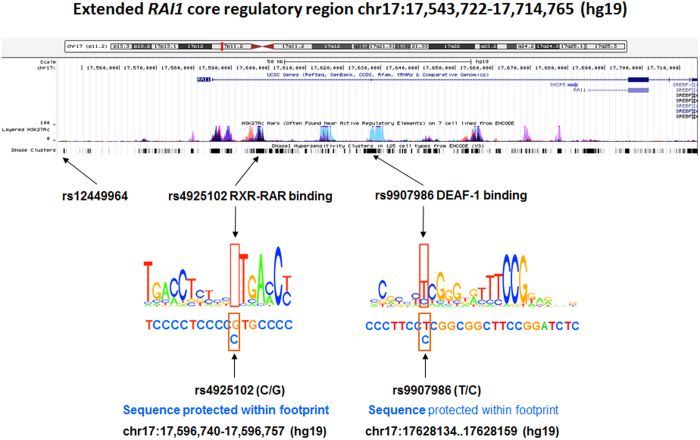
Candidate *RAI1* regulatory variants. (*top*) Screen shot from the UCSC Genome Browser for the indicated region of Chromosome 17 showing the positions of the *RAI1* gene, histone III lysine 27 acetylation and DNase sensitive clusters. (*bottom*) Locations of the “index” SNPs rs12449964, rs4925102 and rs9907986, RXR-RAR and DEAF1 consensus binding sequences, and DNA sequences protected by transcription factor binding in DNase footprint assays as listed in RegulomeDB database (http://www.regulomedb.org/).

Using RegulomeDB, we identified rs4925102 as a RegulomeDB “2b-score” SNP, with evidence for TF binding, a matched TF binding motif, DNase footprint and location within a DNase-sensitive site, that is located in a predicted RXR::RAR_DR5 (Retinoid X Receptor::Retinoic Acid Receptor-Direct Repeat with 5 nucleotide spacing) binding site within the sequence **T**CC**CCTC**C**CC[G/**C**]TG**C**CC**C (Fig. 5). (Nucleic acid residues matching the consensus binding sequence are indicated by bold type). We also identified rs9907986 (which is in complete LD with both rs4636969 and rs8071107) as a “2b-score” SNP, located within the binding site for Deformed Epidermal Autoregulatory Factor-1 (DEAF1). This SNP is predicted to influence the binding of DEAF1 to the sequence **CCTTCC**[**T**/C]**CGGCGGCTTCCGGAT** (Fig. 5), with the SNP **T**-allele more closely matching the consensus sequence, compared to the C-allele. RegulomeDB did not provide information concerning possible functions of SNPs linked to the remaining index SNP, rs12449964.

### Experimental support for the putative functional SNPs in *RAI1* 5′-region

**Figure 6:**
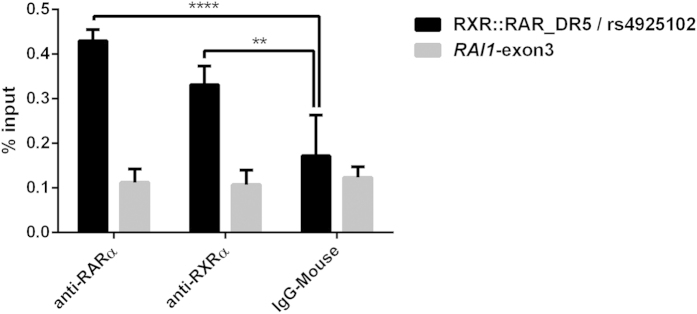
Chromatin immunoprecipitation assays demonstrate binding of RXRα and RARα to the *RAI1* segment containing the putative RXR::RAR_DR5 binding site and rs4925102. Neuronal SH-SY5Y cells were treated with 1 μM all-*trans*-retinoic acid (ATRA) for 135 min prior to cross-linking with formaldehyde and isolation of chromatin. Immunoprecipitation of the cross-linked and fragmented chromatin was performed using mouse anti-RARα (*left*), anti-RXRα (*middle*) antibodies, or normal mouse IgG (*right*). Following purification of immunoprecipitated chromatin and reversal of cross-linking, quantification of target DNA sequences was carried out by real-time PCR using primers designed to amplify a DNA segment containing the putative RXR::RAR_DR5 binding site and rs4925102 (*black bars*) or a DNA segment from *RAI1* exon 3 (*grey bars*). Each measurement was performed in triplicate. Y-axis: amounts of precipitated target DNA sequences expressed as percent (%) of input target DNA sequences in 50 μg chromatin. Statistical significance was assessed by ANOVA followed by post hoc Tukey tests: ****P < 0.0001, **P = 0.0035 (adjusted Tukey test P-values).

To provide experimental evidence for binding of RXR::RAR dimers to the putative RXR::RAR_DR5 binding site in *RAI1* intron 1, we carried out chromatin immunoprecipitation (ChIP) assays using mouse monoclonal antibodies against RXRα or RAR3 transcription factors and PCR primers designed to amplify a 274 bp DNA fragment centered on rs4925102. Consistent with the predicted binding, we observed higher levels of immunoprecipitation of the target DNA fragment with both anti-RXRα and anti-RARα antibodies compared to a control *RAI1* exon 3 DNA fragment in human neuronal SH-SY5Y cells treated with 1 μM all-trans retinoic acid (ATRA) (Fig. 6). Also consistent with predictions, we have obtained direct evidence for the binding of DEAF1 to its putative binding site *RAI1* intron 2 in electrophoretic mobility shift assays (EMSA) and observed a significant increase in luciferase activity driven by the putative DEAF1 binding sequence in heterologous reporter gene expression assays in HEK293T cells. [Li Chen, *et al.*, Functional characterization of novel DEAF1 mutations in clinical whole-exome sequencing of intellectual disability patients and its regulation of the *RAI1* gene. Abstract #387, 64th Annual Meeting of The American Society of Human Genetics, October 10, 2015, Baltimore, MD; manuscript in preparation].

To investigate contributions of the “consensus” index SNPs rs9907986, rs4025102 and rs12449964 to *RAI1* expression in human brain, we plotted normalized ΔΔC_t_ values for *RAI1* mRNA expression in prefrontal cortex and temporal cortex as a function of genotype for each SNP to identify “low-” and “high-expression” alleles (Fig. S4). Each of the SNPs showed nominally significant (P < 0.05) correlations between genotype and *RAI1* mRNA expression in both brain regions, except for SNP rs9907986, which showed a slightly less than significant correlation (p = 0.087) in prefrontal cortex. Assignments of “low-” and “high-expression” alleles for each SNP, the minor allele and its frequency (MAF), and P-values and R^2^ values from single-variable linear regression analysis are listed in Table 2. Multiple linear regression analysis revealed that together rs4925102 and rs9907986 account for 31.1% and 41.1% of the variance of *RAI1* mRNA expression in human prefrontal cortex and temporal cortex, respectively, with only a slight increase observed with the inclusion of rs12449964 (Fig. S5)

## Discussion

In this study, we identified five SNPs located within the 5′- upstream region of the human *RAI1* gene with genotypes that correlate with *RAI1* mRNA expression in Han Chinese samples of prefrontal cortex and temporal cortex. Together these SNPs account for approximately 30% and 40% of the variance in *RAI1* mRNA expression in prefrontal cortex and temporal cortex, respectively. To our knowledge, this is the first report of common, genetically controlled variation in *RAI1* mRNA expression in adult human brain.

Using our R^2^-Δ^2^ method, we showed that the five SNPs that correlated with *RAI1* mRNA expression belong to three SNP “families” with associated “index” SNPs, which together account for approximately 90% of the variance of linear regression R^2^ values for SNPs within the *RAI1* 5′-region. We hypothesize that each SNP family represents one or more variants that directly influence regulation of *RAI1* mRNA expression in brain.

Based on data obtained from RegulomeDB, two of the *RAI1* index SNPs, rs4925102 and rs9907986, are located within transcription factor (TF) binding sites, with alternative SNP alleles predicted to influence TF binding (RegulomeDB score = 2b). Specifically, the index SNP rs4925102 is located within a predicted binding site for dimeric 9-cis retinoic acid receptor (retinoid X receptor, RXR) and all-trans retinoic acid receptor (RAR). The sequence for RXR::RAR binding is located within *RAI1* intron 1 and comprises the DNA sequence **TCCCCTCCCC**G**TGCCCC**, (Chr17:17,596,740-17,596,757; hg19), with nucleic acid residues matching the consensus RXR::RAR_DR5 binding sequence, TGACCTNNNNN[G/C]GACCT^22^, listed in bold type. Rs4925102 [G/C] is located within the 5-nucleotide DR5 spacer, adjacent to the second half-binding motif and is predicted by RegulomeDB to influence the binding affinity of the RXRα/RARα dimer (RegulomeDB score = 2b).

Retinoic acid (RA) signaling plays an important role in cellular proliferation, differentiation, and apoptosis, limb and skeletal defects, abnormal axis patterning of neural tube the central nervous system development^23^, and has been associated with depression^24^, Parkinson’s disease^25^ and autism^26^. *RXR* and *RAR* receptors form a heterodimer that binds retinoic acid and retinoic acid response elements (RARE) to regulate the transcription of target genes^22^. RA is involved in a critical stage of limbic forebrain maturation in early postnatal mice and in a similar stage during the second trimester of prenatal human development^27^. Also, rapid changes in RA signaling in the developing dorsal forebrain coincide with neuronal plasticity and differential regulation of genes encoding proteins that participate in neuronal ligand-receptor interactions and intracellular signaling^28^.

*RAI1* expression has been reported to be increased in the brains of schizophrenia, bipolar disorder, and major depression patients^29^, and *RAI1* to be associated with common neuropsychiatric disorders, including Parkinson’s disease^30^, autism^1^ and schizophrenia^14^. Although *RAI1* was originally cloned from mouse P19 embryonal carcinoma cells exposed to retinoic acid^12^, and RARα has been reported to bind retinoic acid response elements (RAREs) in the *RAI1* promoter^31^, little is known about the effect of RA signaling on *RAI1* expression. This study is the first to show that *RAI1* mRNA expression is controlled in human brain by a common genetic variant located within a binding site for RXRα/RARα retinoic acid receptors. We hypothesize that changes in expression of this dosage-sensitive gene mediated by RA in early brain development may contribute to common neuropsychiatric disorders.

The second *RAI1* index SNP, rs9907986, is located within a consensus binding site for the transcription factor deformed epidermal autoregulatory factor-1 (DEAF1). The predicted sequence for DEAF1 binding is located within *RAI1* intron 2 and comprises the DNA sequence C[**T/C]CGGCGGCTTCCGG**, (Chr17:17628134..17628159; hg19)), with nucleic acid residues matching the consensus DEAF1 binding sequence, TTCGGGNNNTTTCCGG^32^,^33^ listed in bold type. The SNP T-allele generates a sequence closer to the consensus DEAF1 binding sequence, thereby possible increasing TF binding. Interestingly, the sequence TTCG is present within several RAREs^33^ and DEAF1 has been reported to bind to a specific RARE-DR5^33^. DEAF1 bound at these sites, however, is efficiently displaced by RXR::RAR heterodimers^33^.

DEAF1 activates or represses transcription of a variety of genes, including inhibiting its own transcription by binding the motif TTCG, and has previously been shown to be essential for embryonic development in *Drosophila*^34^ and neural tube development in mice^35^. *DEAF1* mRNA and protein are highly expressed in fetal and adult brain, where it is primarily located in the cellular nucleus^33^. DEAF1 contains: i) a SAND (Sp-100, AIRE, NucP41/75, and DEAF1) domain, which mediates multimerization and, in combination with an adjacent zinc finger domain, DNA binding, ii) a nuclear localization signal (NLS) that is essential for localization to the cellular nucleus, iii) a nuclear export signal (NES) sequences, which also functions in multimerization and protein-protein interactions, and iv) a MYND (myeloid translocation protein 8, Nervy, and DEAF1) domain, which mediates protein–protein interactions^36^. Mutations in the SAND domain have been implicated in intellectual disability (ID)^36^, as well as white matter disease and microcephaly^37^. *DEAF1* has also been implicated in major depression, anxiety, suicidal tendencies, and panic disorder though one of its target genes, *5-HT1A*^38^. A SNP (rs6295) within the *5-HTR1A* promoter that interferes with DEAF1 binding disrupts the expression of *5-HTR1A*^39^. Similar to *DEAF1*, mutations in *RAI1* have also been reported to associate with intellectual disability (ID) in a whole-exome sequencing study^40^. The observation that DEAF1 binds to a site within the *RAI1* gene and activates *RAI1* mRNA expression *in vitro* suggests that *RAI1* may be a downstream effector for DEAF1 and genetic differences in the regulation of *RAI1* by DEAF1 may contribute to phenotypic differences observed in SMS and PTLS patients and other common mental disorders.

As shown in Fig. S6, we observed large variation in levels of *RAI1* mRNA expression, which were roughly normally distributed among our Han Chinese brain samples: standard deviation (SD) for prefrontal cortex = 0.9552 ΔCt unit and the SD for temporal cortex = 0.8873 ΔCt unit. Because ΔCt units are measured on the log_2_-scale, these SDs correspond to *RAI1* mRNA levels in the range of mean × [0.52–1.94] and mean × [0.54–1.85] on the linear scale, for *RAI1* mRNA expression in prefrontal cortex and temporal cortex, respectively, for approximately 68% if the population. Likewise, assuming a normal distribution, approximately 32% of the population would be expected to have brain *RAI1* mRNA levels outside of this region.

Large inter-sample variation of mRNA expression has been described for many genes in published PCR and array-based studies of mRNA expression in human brain^41^,^42^,^43^. The large range of measured *RAI1* mRNA levels was surprising, however, since deletion or duplication of the *RAI1* gene, which decreases or increases mRNA expression about 50%^7^,^44^, is pathogenic in both human patients and mice.

Because it is likely that differences in mRNA expression among brain samples are influenced by non-genetic as well as genetic factors, we looked for possible correlations between *RAI1* mRNA expression and sample properties, including: i) gender, ii) age, iii) postmortem interval (PMI) and iv) RNA integrity number (RIN), but found no statistically significant correlations (Fig. S7). Our observation that approximately 30–40% of the variance in *RAI1* mRNA expression can be explained by the genotypes of the three index SNPs, rs9907986, rs4925102 and rs4636969, suggests that genetic differences among individuals may allow *RAI1* mRNA levels to vary by much greater than mean ± 50%. How the human brain can tolerate this degree variance in *RAI1* mRNA expression remains to be explained.

One possibility is that *RAI1* mRNA levels do not always correlate with *RAI1* protein levels. An early study reported that *RAI1* mRNA, but not *RAI1* protein is detected in undifferentiated P19 cells, while both mRNA and protein detected in differentiated cells^12^. The correlation between *RAI1* mRNA levels and protein expression in brain, however, has not yet been systematically examined.

Another possibility is that the differences in *RAI1* mRNA expression observed in this study are actually within the lower end of the normal range of *RAI1* mRNA expression, with the highest and most critical levels of expression attained following induction by retinoic acid or other transcription factors, e.g. DEAF1, during early brain development. The differences in *RAI1* mRNA expression would then depend upon the fold-stimulation of *RAI1* mRNA expression by retinoic acid or other transcriptional activators and may not be as closely correlated with the genotypes of the three index SNPs. Further study will be required to test these hypotheses.

We also examined possible correlations between *RAI1* expression and genotypes of SNPs rs4925102 and rs9907986 in two Caucasian-based brain expression mRNA datasets^45^,^46^, but did not observe statistically significant correlations (results not shown). Statistically significant correlations between rs4925102 or rs9907986 genotypes and *RAI1* mRNA expression were also not observed in two Caucasian-population based lymphoblastoid cell line datasets^47^,^48^ (results not shown). Although it is possible that the lack of correlation between mRNA expression and genotypes for these SNPs reflect population-based differences in regulation of this gene, we suspect that technical differences may be the source for this apparent non-replication. In contrast to our PCR-based assays for quantifying mRNA expression, the above databases used hybridization to microarrays to quantify *RAI1* mRNA levels, a method that is less robust and prone to non-replication^49^,^50^,^51^,^52^ [Song F et al, unpublished observations]. Additional differences, such as the locations of probes used to detect *RAI1* mRNA expression (Exon 3 in our study vs. 3′-UTR in the Colantuoni, C. *et al.*^45^ and Gibbs *et al.*^46^ brain mRNA expression studies) may also have contributed to the lack of replication. Additional PCR-based analyses of *RAI1* mRNA expression in Caucasian brain samples will be required to resolve these issues.

Since most SMS patients harbor large deletions and PTLS patients large duplications at Chr17p11.2 that include *RAI1*, it would be interesting to investigate whether regulatory *RAI1* variants on the “normal” chromosome 17 in these patients modify symptoms associated with these disorders. Specifically, we predict that high expression of *RAI1* from the non-deleted chromosome 17 would ameliorate the effects of *RAI1* deletions in SMS and low expression of *RAI1* from the non-duplicated chromosome 17 would ameliorate the symptoms of *RAI1* deletions in PTLS. Likewise, we predict that low expression of *RAI1* in SMS patients and high expression of *RAI1* in PTLS would exacerbate the symptoms of these disorders. Detailed phenotype and genotype information from sufficiently large SMS and PTLS patient populations will be required to carry out this analysis.

Since *RAI1* is a dosage-sensitive gene that plays a pivotal role in the central nervous system, it would also be interesting to investigate whether genetic variants that regulate *RAI1* expression levels contribute more generally to neuropsychiatric disease susceptibility, including autism spectrum disorder. To our knowledge, there are no reports of statistically significant associations between common *RAI1* genetic variants and human disease, including neuropsychiatric disorders. Because individual *RAI1* variants are likely to make only small contributions to the variance of genetic risk, however, large-scale association studies may be required to detect statistically significant associations. In addition, we suspect that, compared to individual genetic variants, sets of common regulatory and rare coding region mutations in *RAI1* and genes encoding upstream and downstream proteins within *RAI1* signaling pathways will collectively make larger contributions to neuropsychiatric disorders and associations between sets of these genetic variants and disorder phenotypes will be easier to detect.

## Materials and Methods

### Brain samples

Thirty-one frozen samples of dorsolateral prefrontal cortex (Brodmann area 46) and anterior temporal cortex from Han Chinese individuals were obtained from the Chinese Brain Bank Center (CBBC; South-Central University for Nationalities, Wuhan, China). Written consent for tissue donation was obtained from relatives for all samples (on file at CBBC). Use of human autopsy tissue is considered “non-human-subject research” and is Internal Review Board (IRB) exempt under NIH guidelines. The characteristics of our set of temporal cortex samples are presented in Table S2.

### Isolation of genomic DNA and total RNA and synthesis of cDNA

Isolation of genomic (g) DNA and total RNA and preparation of cDNA was carried out as previously described^53^.

### Genotyping

Whole genome genotyping was performed using Illumina HumanOmni1-Quad arrays (Illumina, USA) at Genergy Biotech (Shanghai, China; www.genenergy.cn) and yielded approximately 1.14 × 10^6^ genotypes/sample^53^. Quality control eliminated approximately 300,000 SNPs with minor allele frequencies < 0.01 and 150,000 SNPs with missing data >0.05, yielding approximately 700,000 genotypes per sample. SNPs of interest were examined post hoc for violation of HWE (P < 0.05).

### Genotype imputation

Imputation of *RAI1* was carried out using Impute 2.0^54^ with genotype data from the 1000 Genomes Project (Phase 1) as the reference. All imputed SNPs met the following quality control (QC) criteria: missing rate <0.05; SNP minor allele frequency >0.01; P-value for deviance from Hardy-Weinberg equilibrium >0.001.

### Real-time PCR quantification of *RAI1* mRNA expression

Levels of *RAI1* mRNA temporal and prefrontal cortex samples were quantified by real-time PCR with SYBR-Green (Toyobo) to detect PCR products. The normalized *RAI1* mRNA level in each sample was calculated from three independent measurements of PCR cycle thresholds (Ct’s) for *RAI1* mRNA and mRNAs for one (prefrontal cortex: *ACTB*) or three (temporal cortex) “house-keeping” genes: *β*-*actin (ACTB)*, *cytochrome c-1 (CYC1)* and *hydroymethylbilame synthase (HMBS)*. The sequences of forward and reverse oligonucleotide primers for *RAI1* were: 5′-ATAACCAGCCCGAGTCATGCA-3′ and 5′-TGATGTTTCCTGCGAGGTCTG-3′, respectively. The 91 bp PCR product amplified by these primers spans the end of the 5′untranslated region (UTR) and the first 25 amino acids of the RAI1 coding region within exon 3. Although several *RAI1* mRNA splice variants have been described, northern blot analysis has demonstrated that a single 7668 nucleotide mRNA isoform (NM_030665) is the predominant *RAI1* mRNA detected in all tissues examined, including brain^13^,^14^. Forward and reverse primers for the three house-keeping genes were: i) *ACTB*: 5′-TGTGGCCGAGGACTTTGATTG-3′ and 5′-GTGGCTTTTAGGATGGCA

AGG-3′; ii) *CYC1*: 5′-GAGCACGACCATCGAAAACG-3′ and 5′-CGATATGCC AGCTTCCGACT-3′; iii) *HMBS*: 5′-CCACACACAGCCTACTTTCCA-3′ and 5′-GTACCCACGCGAATCACTCT-3′. Primers were designed using Oligo 6.0 (National Biosciences Inc., Plymouth, MN, USA) and synthesized by Sangon Biotech (Shanghai, China).

The PCR amplification program comprised: i) 1 × [95 °C for 1 min]; ii) 40 × [95 °C for 15 sec, 57 °C for 15 sec, 72 °C for 45 sec]; iii) 1 × [95 °C for 15 sec, 60 °C for 15 sec; 20 min temperature ramp from 60 °C to 95 °C (melting curve), 95 °C for 15 sec]. Following amplification, melting curves of the PCR products were examined to confirm the presence of a single PCR product. Normalized *RAI1* mRNA expression for each brain sample (in ΔCt units) was calculated by subtracting the average Ct obtained for *RAI1* from the average Ct value for *ACTB* (prefrontal cortex) or the geometric mean of average Ct values obtained for each of the three house-keeping genes (temporal cortex). As shown in Fig. S6 normalized *RAI1* mRNA expression levels comprise a nearly Gaussian (i.e., normal) distribution for both prefrontal cortex and temporal cortex samples.

### Statistical and bioinformatics analyses

Correlations between *RAI1* mRNA expression and genotypes of SNPs within the extended or core-*RAI1* locus were quantified by single variable linear regression analysis using the - -assoc command in PLINK (http://pngu.mgh.harvard.edu/~purcell/plink/)^55^ or Minitab 15 (Six Sigma Academy International, L.L.C., State College, PA, USA), providing a P-value and coefficient of determination (R^2^) for each SNP. For selected SNPs, differences in mRNA expression among genotypes were evaluated for statistical significance by ANOVA using SPSS (SPSS Inc., Chicago, IL, USA). To minimize spurious correlations, SNPs with fewer than three heterozygous genotypes among the prefrontal cortex or temporal cortex samples were omitted from the analysis. Plots of pairwise linkage disequilibrium (LD) constants (Δ^2^ = r^2^) for SNPs in the neighborhood of the human *RAI1* gene based upon genotype data for our Han Chinese brain samples were generated using Haploview^56^ (http://www.broadinstitute.org/scientific-community/science/programs/medical-and-population-genetics/haploview/haploview/). Proxy SNP analysis was carried out using SNAP version 2.2^57^ (https://www.broadinstitute.org/mpg/snap/ldsearch.php). Identification of potentially functional SNPs was carried out using data available from RegulomeDB^58^ (http://regulomedb.org/).

### R^2^-Δ^2^ analysis of the contributions of *RAI1* region SNPs to mRNA expression

This method is based on the hypothesis that SNPs located within the neighborhood of a gene correlate with mRNA expression in proportion to their degree of linkage disequilibrium (LD) with *cis*-acting regulatory variants within the same locus. Because the identities of the actual regulatory variants are usually unknown, SNPs that make the largest contributions to the observed variance in linear regression coefficient of determination (R^2^) values for SNPs within the gene locus (termed “index SNPs) are selected and the contributions of all additional SNPs within the locus are assumed to be proportional to the degree of linkage to one or more these SNPs, with additive contributions possible from multiple index SNPs.

The R^2^–value obtained from linear regression analysis of the correlation between normalized log_2_-transformed tissue levels of mRNA and SNP genotype is taken as the measure of the contribution of an individual SNP to mRNA expression. The measure of LD between an “index SNP” and other SNPs in the gene locus is the standard pairwise r^2^ LD constant, which we designate as “Δ^2^” to avoid confusion with “R^2^”.

In mathematical terms, we carry out multiple linear regression analysis of the set of measured R^2^ values for all genotyped and imputed SNPs within a gene locus (the dependent variable) *vs*. sets of pairwise Δ^2^ values calculated between an “index SNP” and all other SNPs in the chromosome region of interest (ROI), with one set for every index SNP (the independent variables). This analysis yields a regression equation:


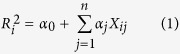


(n = total number of “index” SNPs included in the analysis), where *X*_*ij*_ = pairwise LD constant (Δ^2^_ij_) for the i^th^ SNP and j^th^ index SNP and *α*_*j*_ = regression coefficient reflecting the relative contribution of the j^th^ index SNP to measured R^2^ values, with the values of the coefficients (*α*_*j*_) determined by the regression.

The goodness of fit for each set of index SNPs is determined by regressing measured R^2^ values vs. predicted R^2^ values, which yields an adjusted-R^2^_model_ and a P-value. To avoid bias in the selection of “index” SNPs, an adjusted-R^2^_model_ is calculated for all possible combinations of SNPs in the chromosome ROI, sequentially, for 1, 2, 3 or 4 index SNPs and the smallest combination of SNPs yielding the highest adjusted-R^2^_model_ selected as the best model. To reduce the computational burden of this analysis the list of SNPs within the gene locus is pruned to eliminate SNPs with identical genotypes within the sample set. Also, to avoid mathematical problems arising from colinearity among sets of Δ^2^_ij_ values associated with potential index SNPs, only combinations of SNPs with a maximum variance inflation factor (VIF) < 5 for the associated sets of Δ^2^_ij_ values are accepted as candidates. Finally, because negative contributions to R^2^ are difficult to interpret, only sets of SNPs that yield positive coefficients (αj) for all the terms in the above regression equation are accepted as potential index SNPs.

### Cell culture

Human neuroblastoma-derived SH-SY5Y cells were cultured in 1:1 mixtures of Dulbecco’s Modified Eagle’s Minimum Essential Medium (DMEM) and F12 Medium with 10% (v/v) fetal bovine serum (FBS) and 2 mM L-glutamine and were maintained at 37 °C in a 5% CO_2_ incubator.

### Chromatin immunoprecipitation (ChIP) assays

ChIP assays were performed following the Cold Spring Harbor protocol with minor modifications^59^. SH-SY5Y cells were treated with 1 μM all-*trans*-retinoic acid (ATRA) dissolved in 0.01% dimethylsulphoxide (DMSO) for 135 ± 5 min. The chromatin was digested by micrococcal nuclease (Pierce), as indicated by the manufacturer. Each immunoprecipitation reaction mix contained 50 μg chromatin and 5% of this amount (2.5 μg) chromatin was collected separately to quantify the amount of input target DNA present in each immunoprecipitation reaction mix. One μg mouse anti-RARα antibody (Abcam, ab41934) or anti-RXRα antibody (Sigma, SAB1400249) were used for RARα or RXRα immunoprecipitations, respectively. Immunoprecipitation with 1 μg mouse IgG (Santa Cruz, sc-2025) was used as a negative control. DNA was purified using MinElute reaction cleanup kits (Qiagen) and subjected to real-time PCR using primers designed to amplify appropriate fragments of either the putative RAR::RXR_DR5 binding site containing rs4925102 or the control DNA *RAI1* exon 3 fragment as a non-specific DNA control. The sequences of forward and reverse oligonucleotide primers for rs4925102-RARE were: 5′-AAACCAGGGCATCATTCCTC-3′ and 5′-CCTGACCTTCGACAATGGCTT-3′, respectively. The primers for the control DNA segments were identical to those used to quantify *RAI1* mRNA expression (sequences listed above). All PCR amplifications were carried out in triplicate and the amounts of precipitated target DNA, expressed as percent (%) of input target DNA, were calculated using the formula^60^:


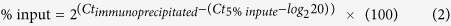


## Additional Information

**How to cite this article**: Chen, L. *et al.* Evidence for genetic regulation of mRNA expression of the dosage-sensitive gene *retinoic acid induced-1 (RAI1)* in human brain. *Sci. Rep.* **6**, 19010; doi: 10.1038/srep19010 (2016).

## Supplementary Material

Supplementary Information

## Figures and Tables

**Table 1:** Pairwise linkage disequilibrium (LD) constants (Δ^2^) of “index” SNPs identified by R^2^-Δ^2^ analysis for *RAI1* mRNA expression in prefrontal cortex and temporal cortex.

		Temporal Cortex “index” SNPs		
Linkage disequilibrium (LD) constants (D2)		rs8071107	rs4925102	rs12449964
	rs4636969	1	0.09	0.121
Prefrontal Cortex “Index” SNPs	rs10401011	0.058	0.735	0.538
	rs12449964	0.133	0.507	1

**Table 2:** Properties of “consensus” index SNPs for *RAI1* mRNA expression in prefrontal cortex and temporal cortex.

SNP	Minor allele	MAF	High expression allele	Low expression allele	Prefrontal cortex R^2^	P-value	Temporal cortex R^2^	P-Value
rs12449964	T	0.1774	T	C	0.254	0.002	0.206	0.006
rs4925102	C	0.1207	C	G	0.241	0.004	0.404	0.0001
rs9907986	T	0.3793	C	T	0.071	0.087	0.121	0.037

Notes: The named alleles are those present in the coding strand; major and minor alleles are defined with respect to the set of brain samples used in this study. Potential functional SNPs with RegulomeDB (http://regulomedb.org/) scores = 2b are listed in bold type.
