# Supplementary material for: Evidence for genetic regulation of mRNA expression of the dosage-sensitive gene retinoic acid induced-1 (RAI1) in human brain
Source: Sci Rep. 2016 Jan 8;6:19010. doi: 10.1038/srep19010 (PMC4705554; doi:10.1038/srep19010)
Supplement: Supplementary Information [file srep19010-s1.doc]

**Evidence for genetic regulation of mRNA expression of the dosage-sensitive gene *retinoic acid induced-1* (*RAI1*) in human brain**

Li Chen^1*^, Yu Tao^1^, Fan Song^1^, Xi Yuan^2^, Jian Wang^4^ and David Saffen^1, 2, 3 *^

^1^ Department of Cellular and Genetic Medicine, School of Basic Medical Sciences, Fudan University

^2^ Institutes of Brain Science, Fudan University

^3^ State Key Laboratory for Medical Neurobiology, Fudan University, Shanghai, China

^4^ Key Laboratory of Exploration and Utilization of Aquatic Genetic Resources, Shanghai Ocean University, Shanghai, Ministry of Education, China

* Corresponding authors: [chenli2008@fudan.edu.cn](mailto:chenli2008@fudan.edu.cn); [saffen@fudan.edu.cn](mailto:saffen@fudan.edu.cn)

Phone: 86-21-54237816

**Supplementary Materials**

**
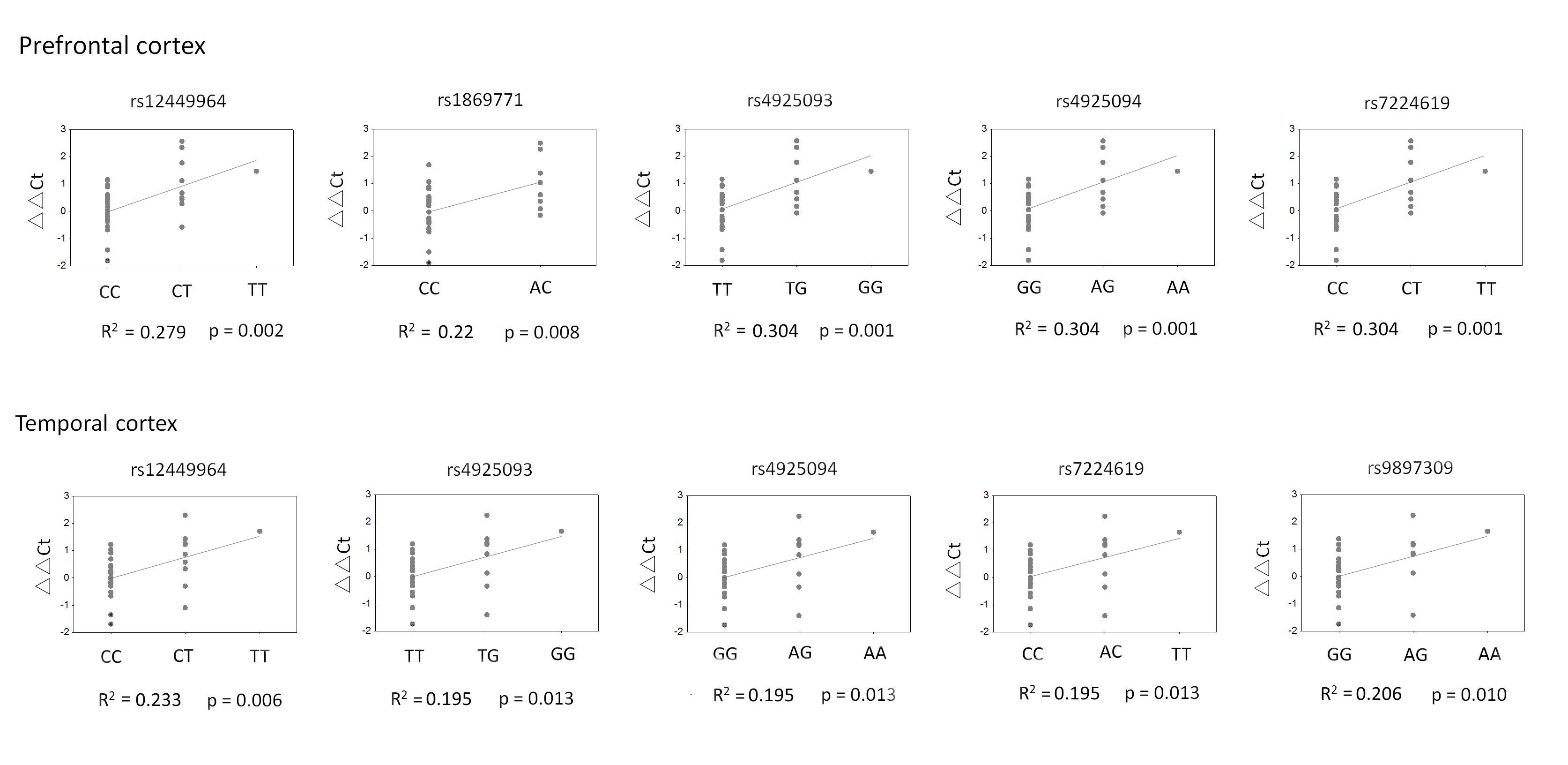
**

Figure S1. Identification of “low-“ and “high-expression” alleles for SNPs that correlate with *RAI1* mRNA expression in Han Chinese prefrontal cortex and/or temporal cortex brain samples. Y-axis: normalized *RAI1* expression [ΔΔCt = (Ct_housekeeping gene(s)_ - Ct*_RAI1_*) - (Ct_housekeeping gene(s)_ – average Ct*_RAI1-LL_*) = average Ct*_RAI1-LL_* - Ct*_RAI1_*]. X-axis: SNP genotypes ordered left-to-right from “low-“ to “high”-expression. Each point within an individual graph represents the average of three independent ΔΔCt measurements in an independent brain sample. R^2^ = linear regression coefficient of determination.


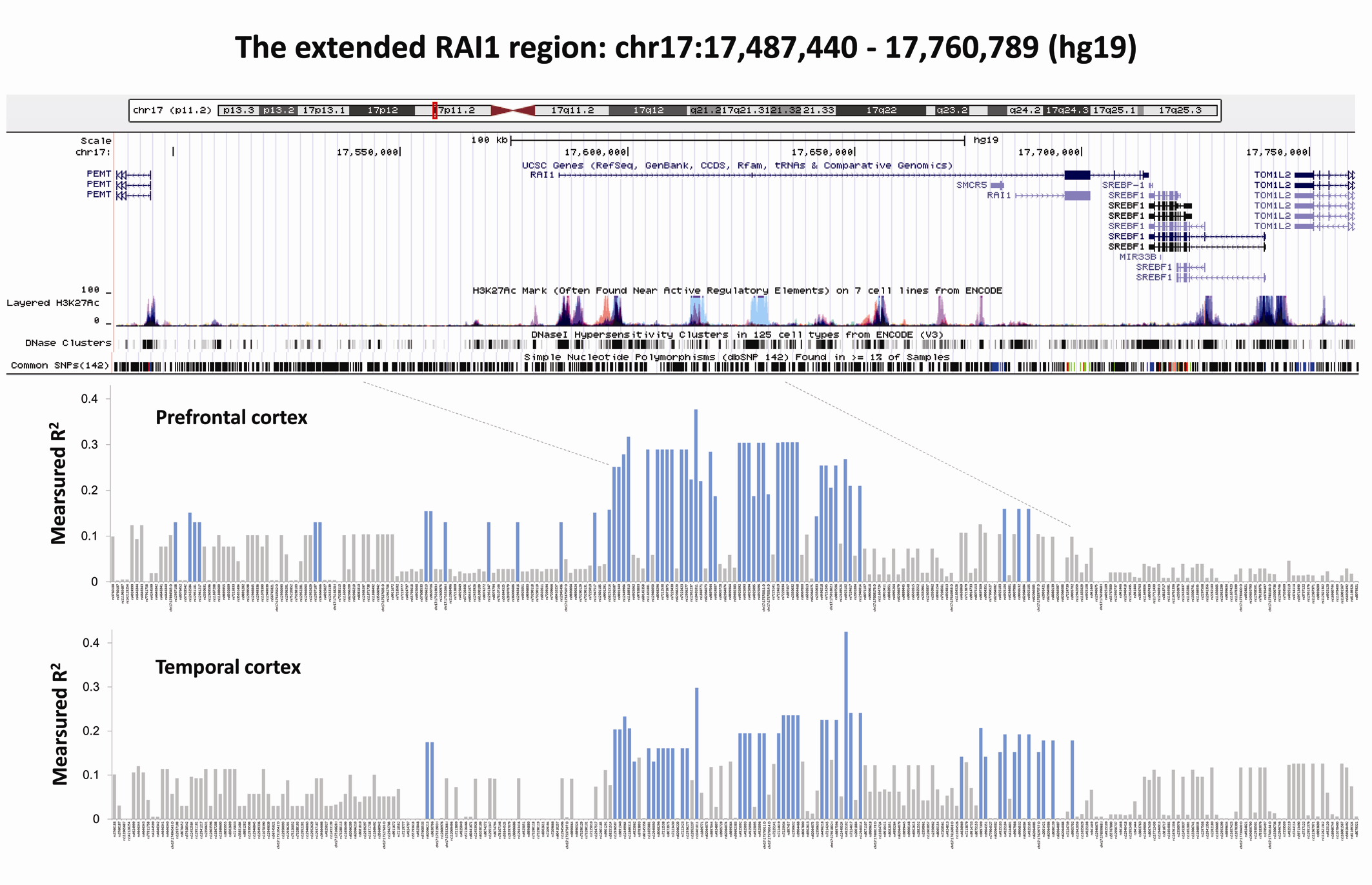


Figure S2. Analysis of 259 genotyped or imputed SNPs within the ~273 kb extended *RAI1* locus for correlations with *RAI1* mRNA expression in prefrontal cortex or temporal cortex. (*top*) Screen shot from the USCS genome browser (http:// genome.ucsc.edu/), showing the locations of protein-coding genes and histone III, lysine 27 acetylation (H3K27Ac) levels detected in cultured cell lines by the ENCODE project. (*bottom*) Plots of R^2^ values determined by single-variable linear regression analysis of *RAI1* mRNA expression vs SNP genotype, with R^2^-values obtained for SNPs showing nominally significant correlations with mRNA expression (P < 0.05; not corrected for multiple testing) indicated by blue bars.


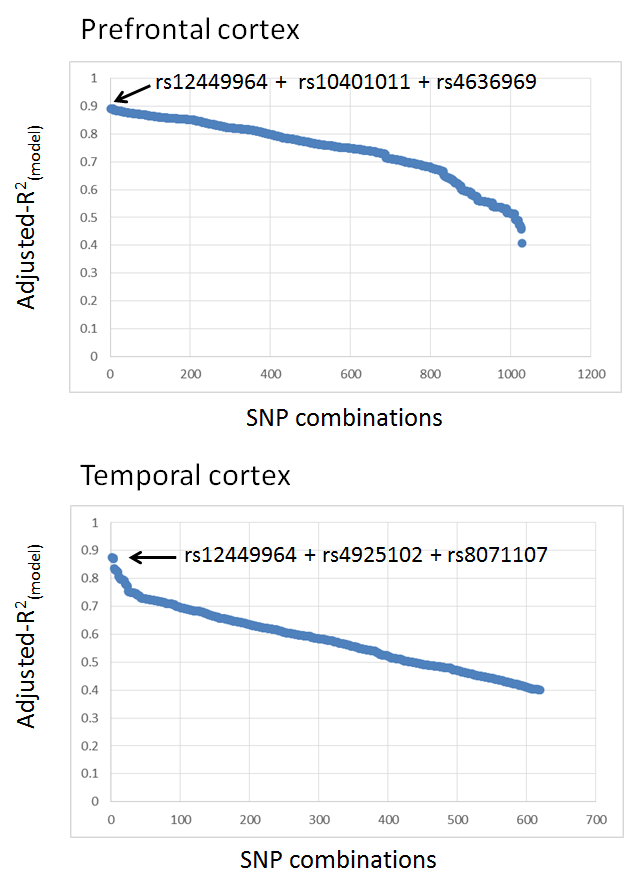


Figure S3. Ranked adjusted-R^2^_model_ values for random combinations of three SNPs from a trimmed list of genotyped or imputed SNPs within the *RAI1* core region (see Methods for details). The best combination of SNPs for prefrontal cortex comprised, rs12449964, rs10401011 and rs4636969, which were subsequently selected as “index” SNPs for further analysis. Likewise, the best combination of SNPs for temporal cortex was identified as rs12449964, rs4925102 and rs8071107 and these were selected as “index” SNPs for subsequent analysis.


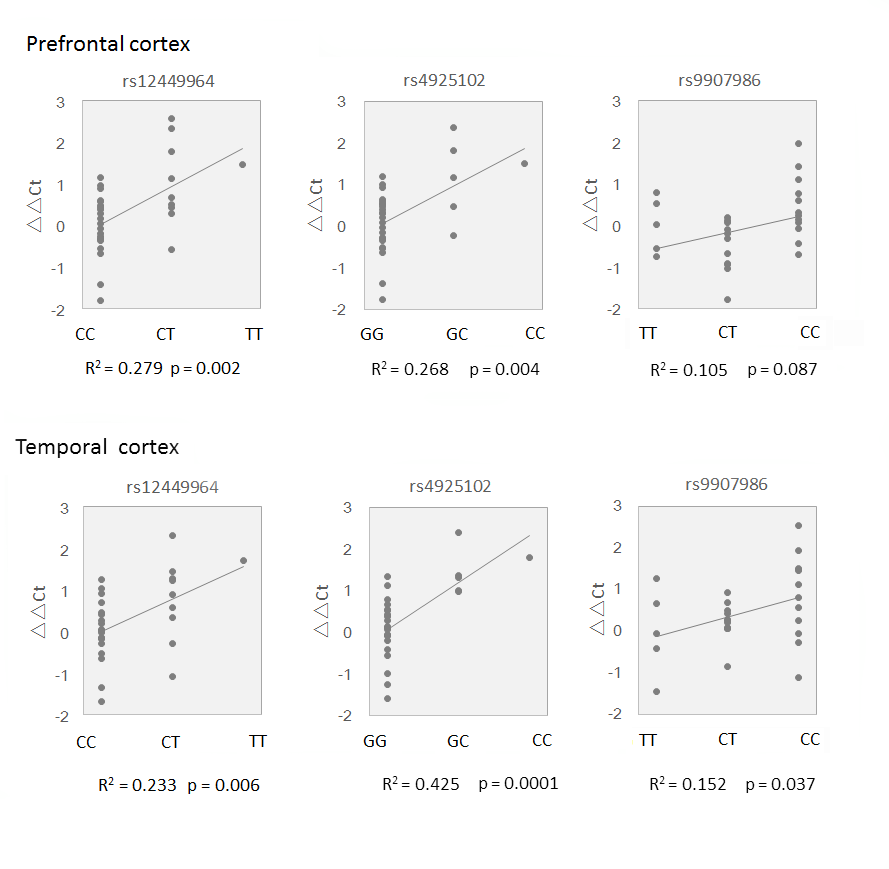


Figure S4. Identification of “low-” and “high-expression” alleles for rs12449964 and the putative functional SNPs rs9907986 and rs4025102. Graphs show the results of single variable linear regression analysis of SNP genotype vs. *RAI1* mRNA expression in Han Chinese prefrontal cortex and temporal cortex brain samples. The units of *RAI1* mRNA expression, ΔΔCt, are defined in the legend of Fig S1. Each point within an individual graph represents the average of three independent ΔΔCt measurements in an independent brain sample.


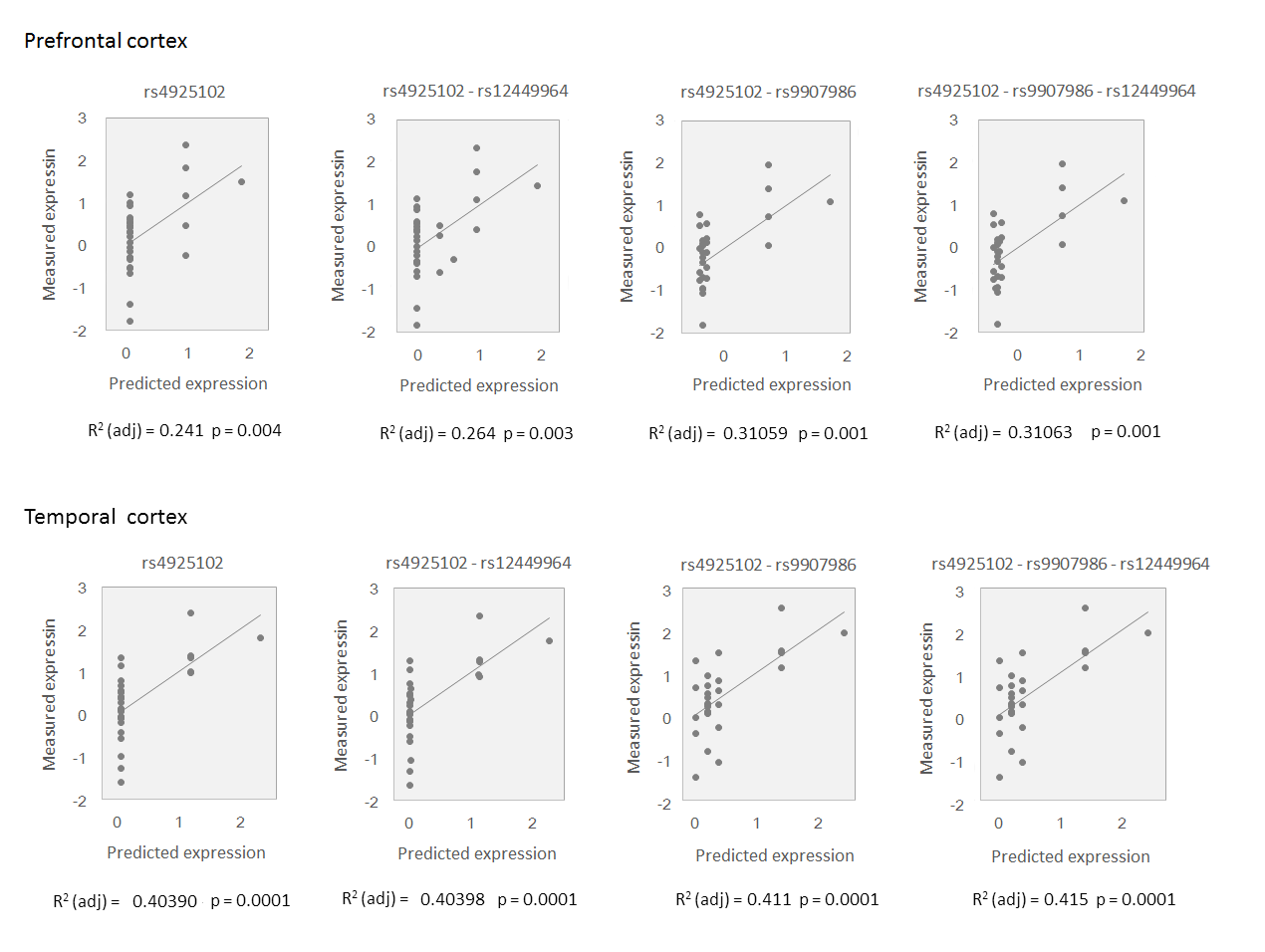


Figure S5. Multiple regression analysis of normalized *RAI1* mRNA expression (△△Ct) vs predicted levels of normalized *RAI1* mRNA expression for rs4925102 genotypes or genotypes combinations for rs4925102/rs12449964, rs4925102/rs9907986, or rs4925102/rs12449964/rs9907986 in human prefrontal cortex and temporal cortex. Y-axis: measured *RAI1* mRNA expression (ΔΔCt units, as defined in Fig S1); X-axis: predicted mRNA expression based on regression equation from single-variable (rs4925102) or multivariable (remaining SNP combinations) linear regression analysis of *RAI1* mRNA expression vs. genotype combinations:


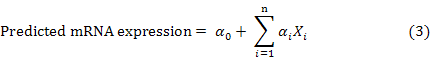


(n = number of SNPs included in the analysis), where *X_i_* = genotype of the i^th^ SNP using the code LL (0), LH (1), and HH (2), and* α_i_* = linear regression coefficient reflecting the relative contribution of the i^th^ SNP to the measured *RAI1* expression level. Each point represents a single genotype (rs4925102) or specific combination of genotypes present within the set of prefrontal cortex and temporal cortex samples.


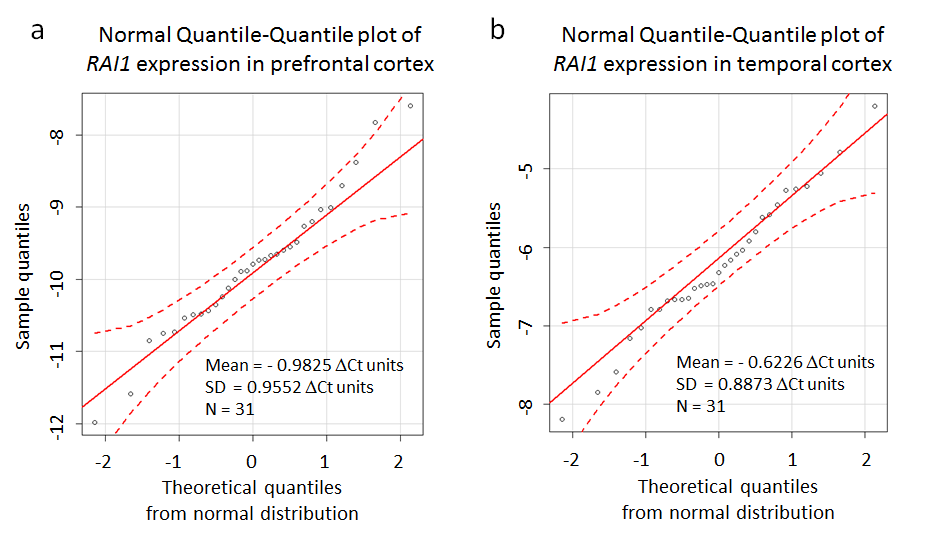


Figure S6. Quantile-Quantile (QQ)-plots demonstrate “Gaussian” distributions of normalized *RAI1* mRNA expression levels in prefrontal cortex and temporal cortex.

a) Normalized levels of *RAI1* mRNA in prefrontal cortex (ΔCt units) were calculated with respect to measured levels of *ATCB* mRNA; b) Normalized levels of *RAI1* mRNA in prefrontal cortex (ΔCt units) were calculated with respect to the geometric mean of measured *ATCB, CYC1* and *HMBS* mRNA expression levels. Dotted lines show 95% confidence intervals for estimation of deviations from normality. SD = standard deviation.


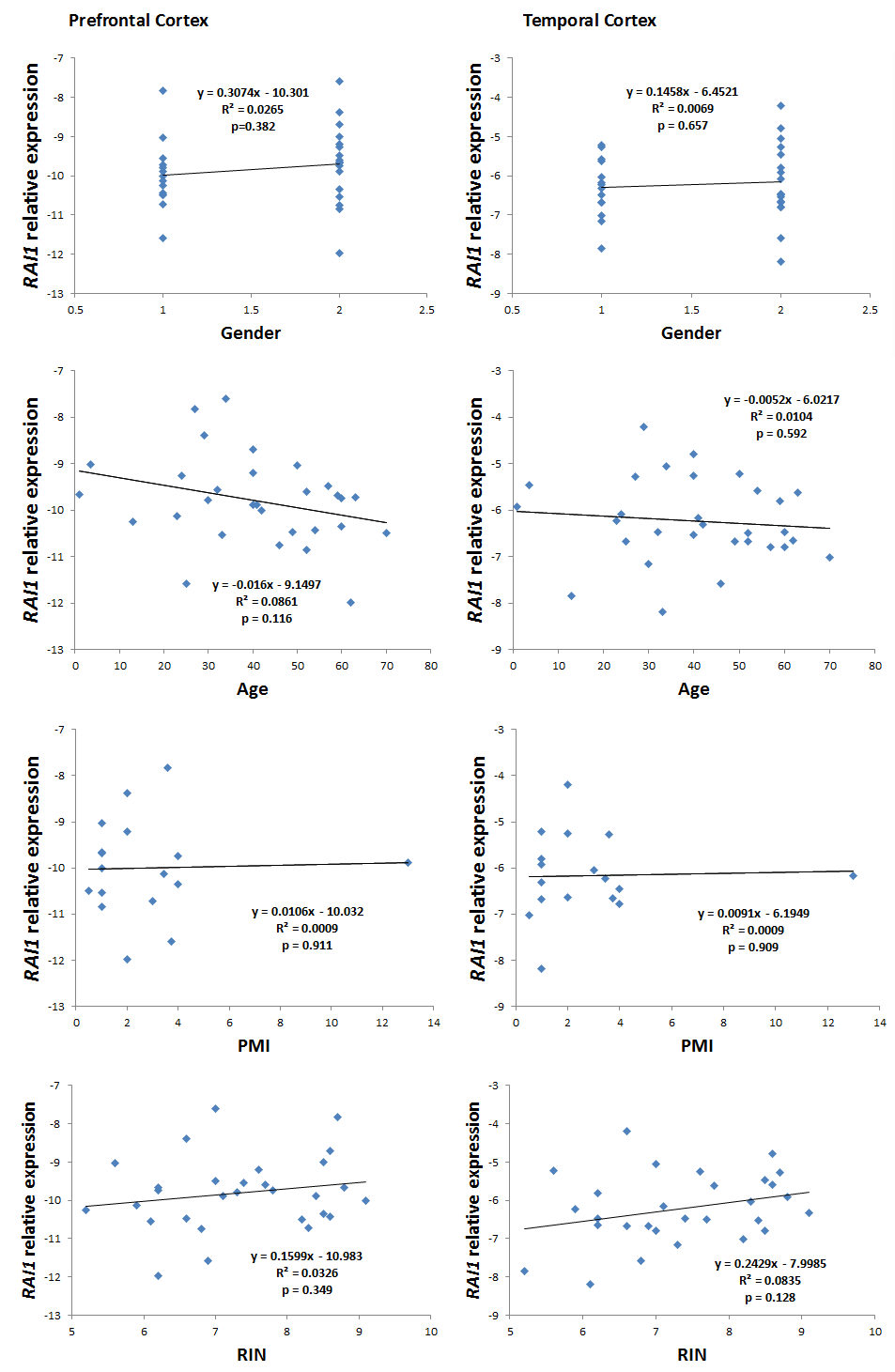


Figure S7. No correlations were observed between gender, age, PMI, or RNA Integrity Number (RIN) values and the relative expression of *RAI1* in prefrontal or temporal cortex. The graphs on the left show the results of linear regression analysis for *RAI1* mRNA expression in prefrontal cortex, the graphs on the right show the results for *RAI1* mRNA expression in temporal cortex.

Table S1. Assignment of “high-“ and “low-expression” alleles based on correlations between *RAI1* mRNA expression in prefrontal cortex or temporal cortex and genotype for *RAI1* 5’-region SNPs. Linear regression P-values are listed, with nominally significant P values (P < 0.05) indicated by **bold** type.

| SNP | rs12449964 | rs1869771 | rs4925093 | rs4925094 | rs7224619 | rs9897309 |
| --- | --- | --- | --- | --- | --- | --- |
| Prefrontal cortex | | | | | | |
| High-expression | *T* | *A* | *G* | *A* | *T* | - |
| Low-expression | *C* | *C* | *T* | *G* | *C* | - |
| Linear regression P-value | **0.002** | **0.008** | **0.001** | **0.001** | **0.001** | 0.051 |
| Temporal cortex | | | | | | |
| High-expression | *T* | *-* | *G* | *A* | *T* | *A* |
| Low-expression | *C* | *-* | *T* | *G* | *C* | *G* |
| Linear regression P-value | **0.006** | 0.186 | **0.013** | **0.013** | **0.013** | **0.01** |

Note: the named alleles are those present in or contiguous with the *RAI1* coding strand.

Table S2. Description of Han Chinese temporal cortex samples

| **Sample No.** | **Gender** | **Age** | **PMI (day)** | **RIN** |
| --- | --- | --- | --- | --- |
|
| 2008274 | F | 29 | 2 | 6.6 |
| 2008281 | F | 59 | 1 | 6.2 |
| 2008295 | M | 50 | 1 | 5.6 |
| 2008298 | F | 40 | 2 | 7.6 |
| 2008306 | F | 62 | 2 | 6.2 |
| 2009050 | F | 24 | N/A | N/A |
| 2009065 | F | 34 | N/A | 7 |
| 2009077 | F | 40 | N/A | 8.4 |
| 2009078 | F | 3.5 | N/A | 8.5 |
| 2009097 | M | 13 | N/A | 5.2 |
| 2009166 | F | 46 | N/A | 6.8 |
| 2009280 | M | 63 | N/A | 7.8 |
| 2009294 | F | 57 | N/A | 7 |
| 2009330 | M | 25 | 3.75 | 6.9 |
| 2009337 | M | 54 | N/A | 8.6 |
| 2009338 | M | 32 | N/A | 7.4 |
| 2010001 | M | 41 | 13 | 7.1 |
| 2010016 | F | 52 | 1 | N/A |
| 2010022 | F | 60 | 4 | 8.5 |
| 2010024 | M | 42 | 1 | 9.1 |
| 2010027 | F | 1 | 1 | 8.8 |
| 2010052 | M | 70 | < 1 | 8.2 |
| 2010056 | F | 40 | N/A | 8.6 |
| 2010066 | F | 52 | N/A | 7.7 |
| 2010085 | M | N.A. | 3 | 8.3 |
| 2010102 | F | 33 | 1 | 6.1 |
| 2010150 | M | 27 | 3.6 | 8.7 |
| 2010154 | M | 23 | 3.45 | 5.9 |
| 2010159 | M | 49 | N/A | 6.6 |
| 2010166 | F | 60 | 4 | 6.2 |
| 2010177 | M | 30 | N/A | 7.3 |

N/A = not available; RIN values for dorsolateral cortex sample: N/A
